# Palmitoylethanolamide as a Supplement: The Importance of Dose-Dependent Effects for Improving Nervous Tissue Health in an In Vitro Model

**DOI:** 10.3390/ijms25169079

**Published:** 2024-08-21

**Authors:** Rebecca Galla, Simone Mulè, Sara Ferrari, Chiara Grigolon, Claudio Molinari, Francesca Uberti

**Affiliations:** 1 Laboratory of Physiology, Department for Sustainable Development and Ecological Transition, University of Piemonte Orientale, UPO, 13100 Vercelli, Italy20029572@studenti.uniupo.it (C.G.);; 2 Noivita S.r.l.s., Spin Off of University of Piemonte Orientale, Via Solaroli 17, 28100 Novara, Italy

**Keywords:** palmitoylethanolamide, supplement, bioavailability, nerve injury, intestinal in vitro model

## Abstract

Palmitoylethanolamide (PEA) is a highly lipophilic molecule with low solubility, making absorption difficult. Recent techniques like micronisation, ultra-micronisation and combining PEA with solvents have improved their bioavailability and stability. Our study analysed particle size differences and absorption kinetics using specific solvents (PEAΩ and PEA DynoΩ) over time (0.5 h–6 h) in a dose-dependent manner (200 mg–1800 mg). The results showed that PEAΩ and PEA DynoΩ achieved 82–63% absorption at 3 h, compared to 30–60% for micronised, ultra-micronised PEA and a commercial product, highlighting the optimal dose range of 300 mg–600 mg. In addition, a 3D model of the peripheral nerve was utilised to explain the efficacy after gut passage and support the most effective dose (300 mg or 600 mg) achieved at the gut level. PEAΩ and PEA DynoΩ, which are associated with better intestinal bioavailability compared to PEA-micronised, PEA ultra-micronised and a commercial product, have allowed not only a reduction in the inflammatory context but also an improvement of peripheral nerve well-being by increasing specific markers like MPZ (26–36% vs. 8–15%), p75 (25–32% vs. 13–16%) and NRG1 (22–29.5% vs. 11–14%). These results highlight the potential of advanced PEA formulations to overcome solubility challenges and maintain in vitro efficacy, modulating peripheral nerve well-being.

## 1. Introduction

Over the past 20 years, the nutrition transition has increased chronic degenerative diseases, mainly due to inflammation. Dietary supplements and foods may help mitigate these hazards [1,2].

One important N-acyl ethanolamine (NAE) is palmitoylethanolamide (PEA), which was first identified in 1957 in egg yolk, soybean and peanut oil [3], and in mammalian tissues in 1965 [4]. Recent investigations have demonstrated its effectiveness in treating neurological illnesses, chronic pain, atopic dermatitis and other disorders [3,5,6].

PEA is a highly lipophilic compound, which gives rise to absorption challenges upon incorporation into a formulation. PEA is practically insoluble in water and poorly soluble in most other aqueous solvents, with the logarithm of its partition coefficient (log P) being >5. The absorption of orally administered PEA is thus likely to be limited by dissolution rate, with the amount absorbed conceivably showing an inverse relation to particle size [7]. The creation of PEA derivatives and prodrugs to enhance their bioavailability and therapeutic benefits has also been extensively investigated [8,9]. An initial step to enhance the absorption of PEA involved a reduction in particle size through a process known as micronisation; this approach results in an increased surface area that facilitates improved absorption kinetics. Micronised pharmaceutical-grade formulations of PEA obtained by jet milling (particle size distribution: 0.8–10 μm) are currently used in human and veterinary medicine for inflammatory, hyperalgesia and allergic disorders. The classical marketed PEA formulations contain (i) unprocessed PEA (frequently referred to as naïve PEA or pure PEA, from 100 μm up to 2000 μm); (ii) micronised PEA (PEA-m, 2–10 μm range) and (iii) ultra-micronised PEA (PEA-um, 0.8–6 μm range) [7]. 

Indeed, formulations of PEA, whether non-micronised, micronised or ultra-micronised, exhibit absorption following oral administration [3,7]. In addition to absorption, pre-systemic metabolism significantly impacts the bioavailability of PEA. Enzymes in both the intestine and liver hydrolyse PEA, resulting in a lipid half-life of about 25 min [10]. Data regarding the distribution of PEA are generally confined to the analysis of blood levels post-oral intake. A study by Petrosino et al. [11] showed a twofold increase in plasma lipid concentrations two hours after 300 mg of micronised PEA ingestion, which returned to baseline levels within 4 to 6 h. Despite only 1% of the compound being metabolised by the body, it exhibits a high diffusion capacity beyond the blood’s aqueous component. This outcome, attributed to PEA’s lipophilic properties, prompts inquiries into its tissue distribution post-oral administration. Notably, Artomonov et al. [12] noted that around 1% of an oral PEA dose was detected in the rat brain, predominantly in the hypothalamus, alongside significant accumulation in the pituitary and adrenal glands. Furthermore, current investigations examine PEA’s position in the endocannabinoid system and its synergistic effects with other cannabinoids, which may lead to novel treatment methods for various inflammatory and neurodegenerative disorders [13]. In particular, PEA has garnered attention for its potential therapeutic benefits in pain management, particularly in neuropathic and somatic pain conditions, due to its anti-inflammatory and analgesic properties [14]. PEA modulates the inflammatory pathways involved in somatic and neuropathic pain, influencing it significantly. Reducing inflammation and its sensitivity to pain by PEA can help manage chronic pain conditions, where inflammation contributes to continued discomfort. Although PEA research on somatic pain is less extensive than neuropathic pain, preliminary studies and clinical reports suggest potential benefits [15]. Based on these recent findings, PEA shows promise in neuropathic and somatic pain management due to its anti-inflammatory and analgesic properties. In addition, a preliminary study showed that PEA could assist with managing perioperative pain and inflammation because of its capacity to activate nuclear receptor peroxisome proliferator-activated (PPAR) receptors and stabilise mast cells [16]. PEA is a unique endogenous compound synthesised within the human body to safeguard cellular integrity in response to damage. Its presence is ubiquitous across various tissues, prominently within the central nervous system, and its activity escalates notably under conditions of illness or injury [1]. Regarding pharmacological effects, PEA is used alone or in combination with antioxidants or analgesics to treat acute and chronic inflammatory disorders due to its anti-inflammatory, immunomodulatory and analgesic capabilities. It regulates both peripheral and central nervous system functions [16]. PEA has anti-inflammatory properties that affect the gut–brain axis, a two-way communication pathway between the central nervous system and the gastrointestinal tract [17,18] through interactions with the endocannabinoid system, which is known to control gastrointestinal motility, secretion and inflammatory responses [19]. Recent research has shown that PEA can improve endocannabinoid signalling at the central nervous system level by blocking the enzyme fatty acid amide hydrolase (FAAH), which breaks down the cannabinoid receptor agonist anandamide [20]. This relationship implies that PEA improves the body’s natural processes for preserving neuronal health, mitigating pain and having direct neuroprotective benefits [18,21]. In addition, several studies have recently explored the therapy of multiple sclerosis, Alzheimer’s disease and diabetic neuropathy due to PEA’s capacity to downregulate mast cell degranulation, decrease neuroinflammation and shield neurons from harm [22]. Moreover, different clinical applications have demonstrated the involvement of PEA in peripheral neuropathic pain, musculoskeletal pain and palliative care [23]. In 2017, an early clinical trial meta-analysis suggested that PEA could be clinically useful in treating chronic pain, estimated to affect 38% of people worldwide [24]. An important role exerted by PEA is related to its ability to reduce the activity of pro-inflammatory enzymes such as cyclooxygenase (COX), eNOS and iNOS, reducing mast cell activation [24]. Rising levels of PEA led to higher concentrations of cannabinoids, which then regulated elements related to stress, neuroinflammation and cognition [25]. The diverse impacts of PEA arise from its distinct mechanism of action, which influences various pathways at different locations [26]. Primarily, it targets the PPAR-α. Additionally, PEA affects novel cannabinoid receptors, namely G-protein-coupled receptor 55 (GPR55) and G protein-coupled receptor 119 (GPR119). GPR55 has recently been reported to be involved in addressing inflammation [27]. Moreover, it indirectly activates cannabinoid receptors 1 and 2 (CB1 and CB2) by inhibiting the degradation of the endocannabinoid anandamide (AEA), resulting in the “entourage effect” [3]. CB1 is found in the peripheral nervous system and almost all mammalian tissue, while CB2 is expressed at a lower level in the brain but is mainly expressed in astrocytes and microglia [27]. 

Furthermore, PEA alleviates pain by decreasing the sensitivity of TRPV1 channels, achieved through the synergy of PPAR-α activation and potential allosteric regulation. Professor Rita Levi Montalcini characterised this process as autacoid local inflammatory antagonism (ALIA), which blocks mast cell activation [28,29]. PEA lowers the production of pro-inflammatory cytokines and modifies the immunological response in the peripheral nerve system, which is frequently dysregulated in neuropathic pain. Furthermore, by stabilising mast cells and reducing the migration of immune cells to the site of nerve damage, PEA lessens hyperalgesia or enhanced sensitivity to pain [18,21]. In addition, PEA continues to demonstrate promise in the peripheral nervous system (PNS) for neuropathic pain relief. Indeed, in a more recent clinical study, ultra-micronised PEA significantly reduced pain and improved nerve function in patients with diabetic neuropathy, demonstrating the compound’s efficacy as a safe and effective adjuvant medication [30]. 

**Figure A1:**
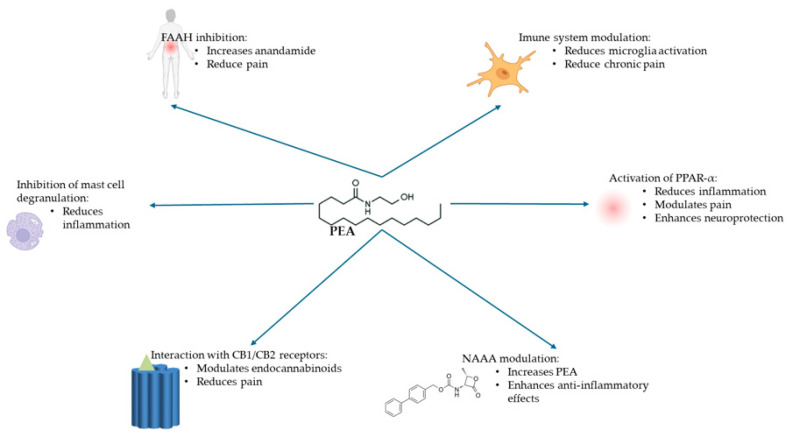
General schematic figure on the mechanism of action and enzymatic interferences of PEA.

The therapeutic benefits of PEA are generated through multiple mechanisms of action (Figure A1 in Appendix A); however, the natural levels of endogenous PEA are generally insufficient to counteract the chronic allostatic load observed in chronic inflammatory disorders. As a result, the administration of exogenous PEA becomes a viable therapeutic strategy to restore endogenous levels and promote body homeostasis [31]. Also, the absorption rate is constrained by various factors, such as the rate at which the substance dissolves and the presence of an aqueous barrier in the gastrointestinal lumen. These factors are affected by the lipophilicity and particle size of PEA. After absorption, PEA undergoes quick metabolism and elimination, resulting in a relatively short half-life. The levels of PEA in human plasma return to their baseline values within two hours of ingestion [11]. PEA plays a crucial role as an anti-inflammatory, analgesic and neuroprotective agent by targeting various molecular pathways in the central and peripheral systems [32,33]. In addition, PEA also exerts a crucial function in decreasing oxidative stress. For instance, when neurons are subjected to terbutyl hydroperoxide-induced stress, the presence of PEA results in a lesser increase in markers of lipid peroxidation [17].

Based on this evidence, it is important to elucidate the significance of PEA’s preparation method and dosage in enhancing its therapeutic effectiveness. Due to poor plasma concentrations caused by several variables, the therapeutic effectiveness of PEA is reduced, and bigger dosages are needed to provide the intended results [34]. However, high dosages may cause unanticipated side effects and unpredictability in patient responses, which are unacceptable in therapeutic settings [1]. Regarding the dosage, it is important to remember that the guidelines suggest starting with low doses (200–400 mg) for new therapy or at-risk patients, medium doses (600–1200 mg) for balanced efficacy and safety and higher doses (1500–1800 mg) when enhanced efficacy is required, with careful monitoring to manage risks. Simplified dosing regimens are preferred for patient adherence [35,36]. 

To improve PEA’s bioavailability, recent research has concentrated on creating innovative formulations combining PEA with some natural extract [37] or using new delivery strategies [38]. For example, novel delivery methods such as co-crystals to improve the solubility and stability of PEA, nanoparticles and lipid-based carriers have also been researched to prolong the release. This has helped to further optimise PEA’s dosage and reduce the requirement for high concentrations. These developments highlight how crucial it is to solve the bioavailability problem to fully realise PEA’s medicinal potential by optimising PEA’s dose and administration form [4]. Ultra-micronised PEA dramatically increases bioavailability compared to non-micronised PEA, which enhances the therapeutic results, especially when treating chronic pain and inflammation [4]. At the same time, the development of PEA-loaded nanoparticles and co-crystals may provide sustained release patterns, extending the duration of their therapeutic benefits [22].

This study will determine how PEA’s manufacturing process and dose influence its therapeutic effectiveness. The goal is to investigate how different PEA formulations and dosages affect intestine absorption and assess peripheral effects, emphasising enhancing peripheral nerve health. Since a previous study indicated that PEA’s bioavailability and therapeutic effectiveness are substantially impacted by its formulation and dose, this study intends to provide more insight into these dynamics by discovering the ideal settings for maximising PEA’s therapeutic effects. This study, through the meticulous examination of various formulations and doses, intends to clarify the critical impact these aspects play in improving the therapeutic use of PEA for treating neuropathy.

## 2. Results

### 2.1. Analysis of Absorption Rate and Integrity in an In Vitro Intestinal Barrier Model

**Figure 1:**
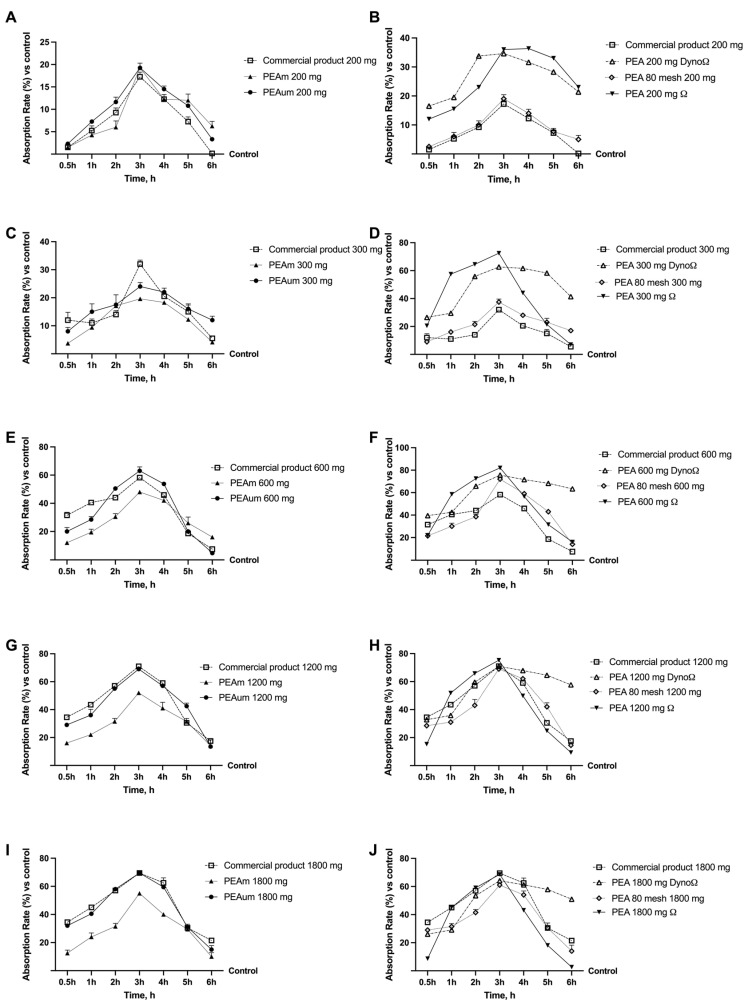
Dose–response (200 mg–1800 mg) and time–course studies of the absorption rate of different forms of PEA. (**A**,**B**) Absorption rates of all PEA forms at the concentration of 200 mg; (**C**,**D**) absorption rates of all PEA forms at the concentration of 300 mg; (**E**,**F**) absorption rates of all PEA forms at the concentration of 600 mg; (**G**,**H**) absorption rates of all PEA forms at the concentration of 1200 mg; (**I**,**J**) absorption rates of all PEA forms at the concentration of 1800 mg. All absorption analyses were performed after 0.5 h to 6 h of treatment. PEAm = PEA micronised; PEAum = PEA ultra-micronised; PEA 80 mesh = PEA 80 mesh; PEAΩ = PEA 80 mesh dissolved in Ω solvent; PEADynoΩ = PEAΩ = PEA 80 mesh dissolved in Ω solvent included in Dyno. Data are mean ± SD of five independent experiments performed in triplicates vs. control values (0% line, n = 5 independent observation per treatment group and each sample was run in triplicate).

The low bioavailability and poor absorption of PEA have been repeatedly reported in the literature [27]. Therefore, an initial set of experiments was conducted on different PEA forms to verify if this limitation is due to the concentration used in addition to the preparation. In Figure 1, all the absorption data for the different forms of PEA at different concentrations have been reported. The data showed that all forms of PEA at the 200 mg concentration possess a higher absorption rate than the control (*p* < 0.05), but not all of them are better than the commercial product (Figure 1A,B). Indeed, only PEAum, PEA 80 mesh, PEAΩ and PEADynoΩ showed a higher absorption rate than the commercial product (*p* < 0.05). The mechanisms behind these results can be attributed to the excipients in the commercial product, suggesting the modulation of PEA’s uptake depends on them, in line with the water dispersion and bioavailability reported in the literature [25]. In contrast, for the 300 mg concentration (Figure 1C,D), only PEA 80 mesh, PEAΩ and PEADynoΩ showed a higher absorption rate than the commercial product (*p* < 0.05), but all forms are still higher in absorption rate than the control (*p* < 0.05). Regarding the 600 mg concentration (Figure 1E,F), all forms of PEA showed a higher absorption rate than the control, while PEAum, PEAΩ and PEADynoΩ were found to have a higher absorption rate compared to the commercial product (*p* < 0.05). The forms of PEA administered at a concentration of 1200 mg showed that only PEAΩ possessed a higher absorption rate than the commercial product (*p* < 0.05); this might suggest that the technology used for preparation may improve absorption at the intestinal level (Figure 1G,H). However, all other forms of PEA showed a higher absorption rate than the control (*p* < 0.05). Similarly, even after stimulation with the forms of PEA at the concentration of 1800 mg (Figure 1I,J), all forms of PEA showed a higher absorption rate than the control (*p* < 0.05), but none were higher than the commercial product. In summary, the data indicate that across all concentrations of PEA forms, only PEAΩ and PEADynoΩ at 300 and 600 mg showed peak absorption after 3 h of treatment, in comparison to the commercial product, which had absorption rates of around 42% and 31% (PEA PEADynoΩ 300 and 600 mg vs. the commercial product) and 50% and 21% (PEAΩ 300 and 600 mg vs. the commercial product).

**Figure 2:**
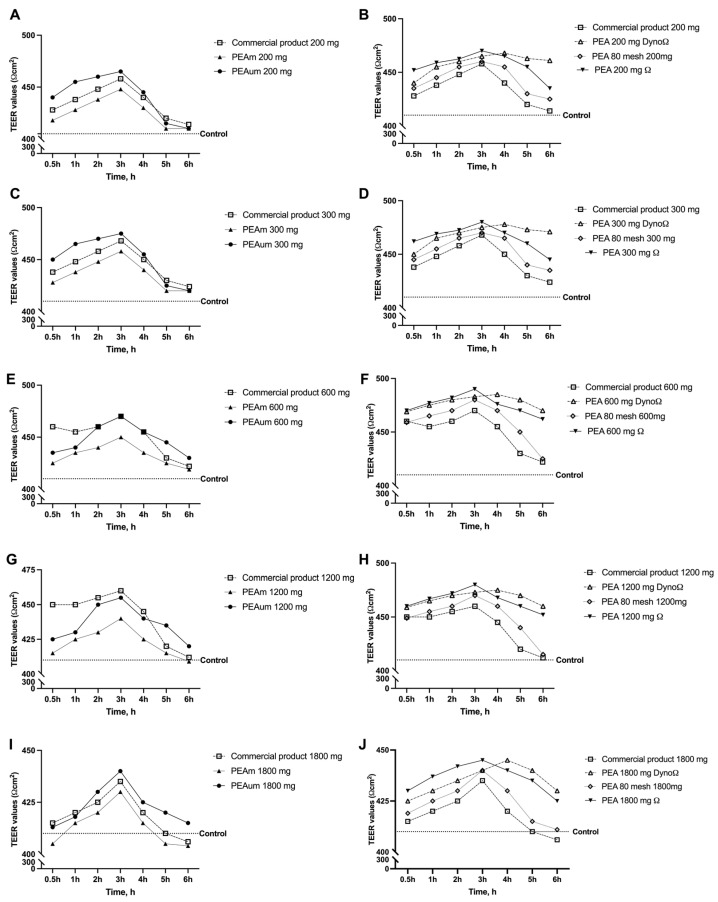
Dose–response (200 mg–1800 mg) and time–course studies of intestinal barrier integrity on Transwell^®^ Corning^®^ Costar^®^, Merck Life Science, Rome, Italy) by TEER measurements. (**A**,**B**) TEER values of all PEA forms at the concentration of 200 mg; (**C**,**D**) TEER values of all PEA forms at the concentration of 300 mg; (**E**,**F**) TEER values of all PEA forms at the concentration of 600 mg; (**G**,**H**) TEER values of all PEA forms at the concentration of 1200 mg; (**I**,**J**) TEER values of all PEA forms at the concentration of 1800 mg. All integrity analyses were performed after 0.5 h to 6 h of treatment. The abbreviations are the same as those used in Figure 1. Data are mean ± SD of five independent experiments performed in triplicates vs. control values (0% line, n = 5 independent observation per treatment group and each sample was run in triplicate).

Additional experiments were conducted to obtain important information on intestinal integrity using an in vitro intestinal barrier. The transepithelial electrical resistance (TEER) analysis performed in this study, from 30 min to 6 h, showed that all types of PEA, when administered at a concentration of 200 mg (Figure 2A,B and Table A1), were effective at preserving the structural integrity of epithelial cells and facilitating the movement of ions through the paracellular pathway. In particular, PEAum, PEA 80 mesh, PEAΩ and PEADynoΩ could maintain the integrity of the intestinal barrier better than all other forms of PEA, including the commercial product (*p* < 0.05). A similar trend is shown in Figure 2C,D (Table A2), in which the cell monolayer integrity data are shown after treatment with all forms of PEA at the concentration of 300 mg. Even at this concentration, all forms of PEA maintained intestinal integrity better than the control. At the same time, PEAum, PEA 80 mesh, PEAΩ and PEADynoΩ were able to maintain intestinal barrier integrity in a better-performing manner than the commercial product. Furthermore, Figure 2E,F illustrate the TEER values post-administration of various formulations of PEA at a dosage of 600 mg (reported also in Table A3). Notably, while the various forms of PEA do not exhibit detrimental effects on intestinal integrity, only PEAΩ and PEADynoΩ have enhanced benefits compared to the commercial product (*p* < 0.05). Moreover, after exposure to a dosage of 1200 mg (Figure 2G,H and Table A4), it was observed that there was no impairment to the structural soundness of the intestinal monolayer across all variations of PEA, with levels consistently exceeding those of the control group (*p* < 0.05). Furthermore, it was noted that PEAΩ and PEADynoΩ exhibit superior preservation of cellular integrity compared to the commercial product and other forms of PEA that were examined (*p* < 0.05). In conclusion, the impacts of various forms of PEA administered at a concentration of 1800 mg (Figure 2I,J and Table A5) were assessed. PEAm tends to uphold the integrity of the cellular monolayer at this particular dosage, albeit not reaching the control values across the entire span of treatments examined. Conversely, some variations outperform the commercial product by ensuring integrity values surpass even the control values (*p* < 0.05); this pattern has been identified in PEAum, PEA 80 mesh, PEAΩ and PEADynoΩ. 

**Figure 3:**
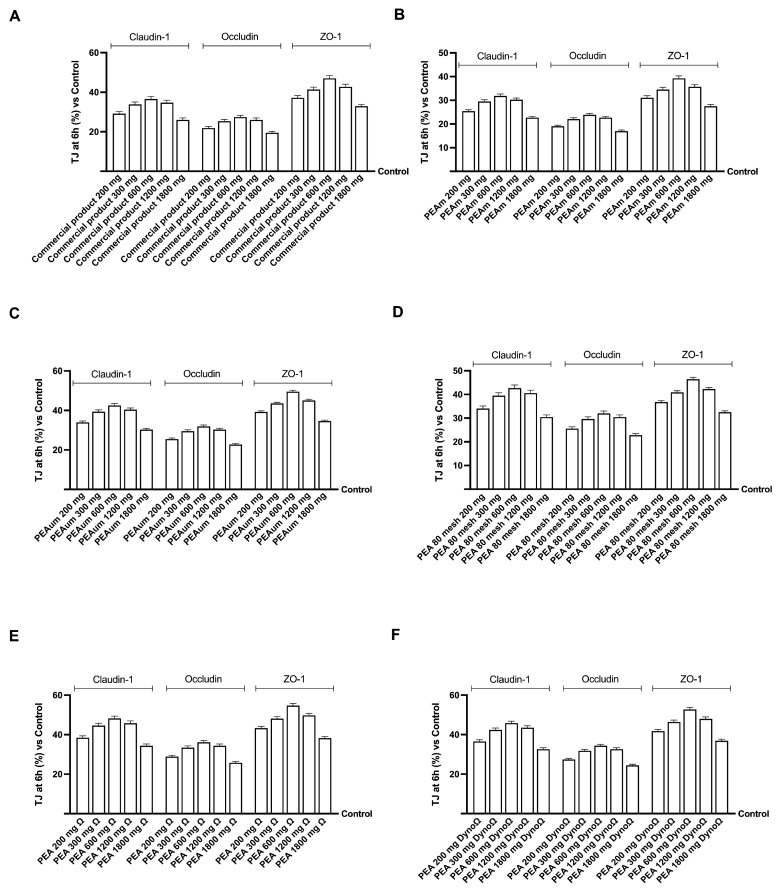
Analysis of intestinal barrier integrity on Transwell^®^ by TJ analysis. (**A**) TJ analysis of all the commercial product concentrations; (**B**) TJ analysis of all PEAm concentrations; (**C**) TJ analysis of all PEAum concentrations; (**D**) TJ analysis of all PEA 80 mesh concentrations; (**E**) TJ analysis of all PEAΩ concentrations; (**F**) TJ analysis of all PEA DynoΩ concentrations. All integrity analyses were performed after 6 h of treatment. The abbreviations are the same as those used in Figure 1. Data are mean ± SD of five independent experiments performed in triplicate vs. control values (0% line, n = 5 independent observation per treatment group and each sample was run in triplicate).

After TEER analysis, the protein levels of the tight junctions (TJ) responsible for the bonds between the cells that form the monolayer were determined to confirm the integrity of the cell monolayer. As can be seen in Figure 3, all forms of PEA in the concentrations tested can increase the levels of the three TJs analysed (Claudina-1, Occludin, Zo-1) compared to the control (*p* < 0.05), confirming their safety at the intestinal level. Among all the forms tested, PEA 80 mesh, PEAΩ and PEADynoΩ showed a beneficial effect similar to that of the commercial product. In addition, PEAΩ proved to be the best form in firm joints, as it had a greater biological effect than the commercial product and all other PEA forms tested. All these TJ data indicate that among all the concentrations of PEA forms tested, the most effective ones were PEAΩ and PEADynoΩ at 300 and 600 mg doses. More specifically, after being treated with PEADynoΩ 300 mg, the levels of claudin, occludin and ZO-1 showed an increase of around 24%, 21% and 14%, respectively, compared to the products available on the market. Likewise, PEADynoΩ at 600 mg also raised TJ levels by about 25% (claudin), 23% (occludin) and 16% (ZO-1) in comparison to the commercial product. PEAΩ at 300 and 600 mg doses also managed to elevate TJ levels by approximately 20% and 22% (claudin), 25% and 27% (occludin) and 11% and 13% (ZO-1), respectively, when compared to the commercial product.

To summarise, from these initial absorption and intractability studies, it can be assumed that all forms of PEA are well tolerated in the intestine, even when administered at different doses. In particular, a dose-dependent effect of all forms of PEA can be assumed, reaching the maximum effect at concentrations of 300 mg, 600 mg and 1200 mg. In contrast, doses of 200 mg and 1800 mg seem to be the minimum and maximum doses to achieve any kind of effect at the cellular level. In addition, the PEA samples examined and included in the dose range of 300 mg and 1200 mg showed similar effects at the intestinal level. However, these hypotheses were also tested in the next experiments to determine whether all forms of PEA have a beneficial effect once they cross the intestinal barrier and reach the bloodstream.

### 2.2. Effects of the Different PEA Forms on 3D EngNT Co-Cultures

More precisely, the nerve tissue subjected to 200 ng/mL glial growth factor (GGF) treatment exhibited a decrease in cell viability in comparison to the control level (*p* < 0.05, presented as 0% in the figure legend). Conversely, the application of varying doses of different forms of PEA resulted in a dose-dependent restoration of cell viability following the damage induced by 200 ng/mL GGF, showing values surpassing those of the control. Regarding cell viability, PEAΩ and PEADynoΩ proved to be the most efficacious among the tested forms at all concentrations assessed (*p* < 0.05). The data presented in Table 1 show that the dosages of 300, 600 and 1200 mg stood out as the most effective at the peripheral level among the various forms of PEA. Notably, PEAΩ and PEADynoΩ were the top performers at these dosages, with cell viability increasing by approximately 1.4-fold and 1.2-fold at the 300 mg dosage, approximately 2.3-fold and 2.1-fold at the 600 mg dosage and approximately 2.5-fold and 2.3-fold at the 1200 mg dosage compared to the commercial product.

**Figure 4:**
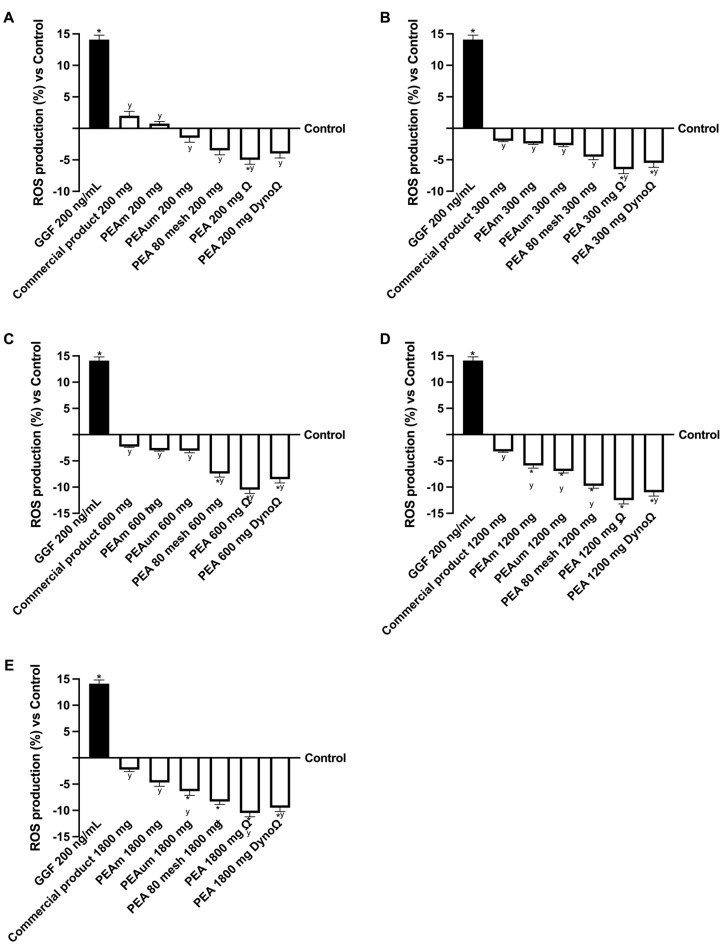
Analysis of the PEA formulation effects under PNI conditions. (**A**) ROS production after treatment with all PEA forms at the concentration of 200 mg; (**B**) ROS production after treatment with all PEA forms at the concentration of 300 mg; (**C**) ROS production after treatment with all PEA forms at the concentration of 600 mg; (**D**) ROS production after treatment with all PEA forms at the concentration of 1200 mg; (**E**) ROS production after treatment with all PEA forms at the concentration of 1800 mg. GGF = glial growth factor 2; the other abbreviations are the same as those reported in Figure 1. Data are mean ± SD of five independent experiments performed in triplicates vs. control values (0% line, n = 5 independent observation per treatment group and each sample was run in triplicate). * *p* < 0.05 vs. control; ^y^ *p* < 0.05 vs. GGF 200 ng/mL.

A similar pattern to viability was noted concerning the generation of reactive oxygen species (ROS) (Figure 4). The nerve tissue exposed to damage from 200 ng/mL GGF displayed a higher ROS production than the control level (*p* < 0.05), which was notably decreased following treatment with various concentrations of PEA forms in comparison to the damage (*p* < 0.05). Specifically, PEAΩ and PEADynoΩ exhibited a more substantial reduction in ROS production at all evaluated concentrations than all other tested PEA forms (*p* < 0.05). In conclusion, this initial analysis at the peripheral level suggests that different forms of PEA may impact the ultimate target with varying effectiveness, influenced partly by their particle size but primarily by the administered dose. Indeed, the optimal dosages that effectively mitigated the overproduction of ROS caused by the damage induced by GGF at a concentration of 200 ng/mL are 300 mg, 600 mg and 1200 mg for all variants of PEA examined, with a notable emphasis on PEAΩ and PEADynoΩ. Samples evaluated at a dose of 300 mg demonstrated antioxidant and decreased oxidative stress effects equivalent to the 1200 mg dosage in the 300–1200 mg range. Specifically, among all the types of PEA, PEAΩ and PEADynoΩ stood out at these doses, showing a significant reduction in ROS production. This reduction correlated to PEAΩ and PEADynoΩ amounting to approximately 1.6-fold and 1.75-fold at a 300 mg dosage, 3.5-fold and 2.7-fold at a 600 mg dosage and 2.9-fold and 2.4-fold at a 1200 mg dosage compared to the commercial product. When PEA is used at dosages higher than these, research has shown that its effectiveness declines significantly.

**Figure 5:**
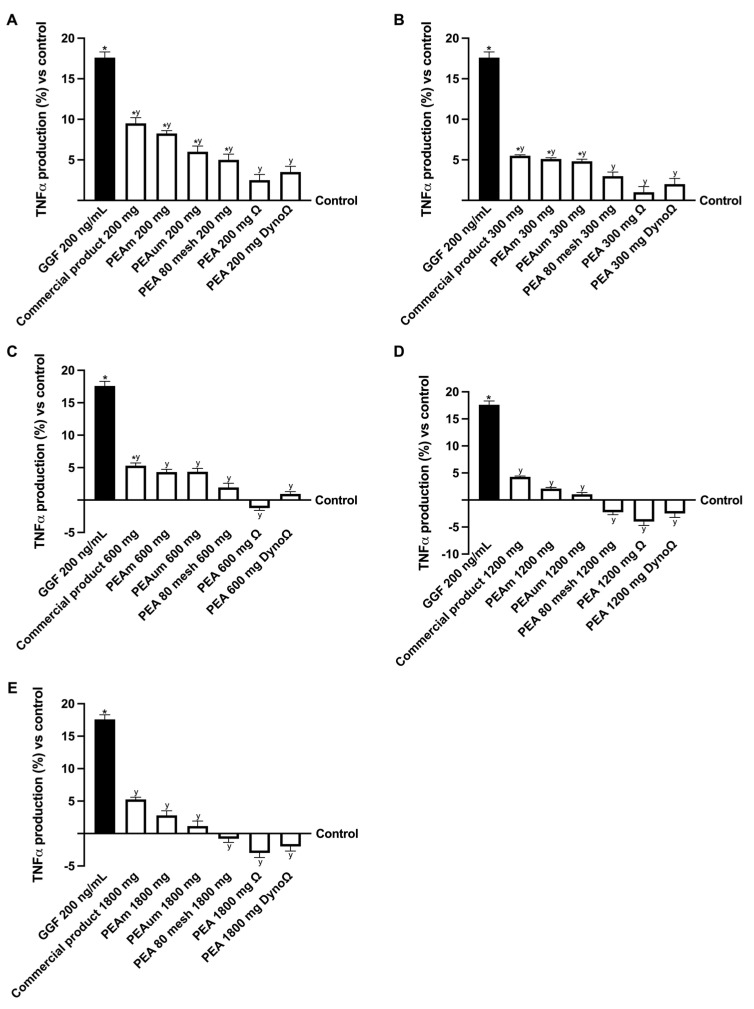
Analysis of the PEA formulation effects under PNI conditions. (**A**) TNFα production after treatment with all PEA forms at the concentration of 200 mg; (**B**) TNFα production after treatment with all PEA forms at the concentration of 300 mg; (**C**) TNFα production after treatment with all PEA forms at the concentration of 600 mg; (**D**) TNFα production after treatment with all PEA forms at the concentration of 1200 mg; (**E**) TNFα production after treatment with all PEA forms at the concentration of 1800 mg. The abbreviations are the same as those reported in Figure 4. Data are mean ± SD of five independent experiments performed in triplicates vs. control values (0% line, n = 5 independent observation per treatment group and each sample was run in triplicate). * *p* < 0.05 vs. control; ^y^ *p* < 0.05 vs. GGF 200 ng/mL.

Moreover, to confirm the efficacy of PEA formulations post-intestinal transit in eliciting a peripheral recuperative impact, an examination was conducted on one of the major inflammatory markers associated with neuropathy, namely tumour necrosis factor α (TNFα) (Figure 5). It can be postulated that the advantageous characteristics of the various forms and strengths of PEA assessed may counterbalance the inflammatory cascade incited by GGF at 200 ng/mL. Specifically, pretreatment with GGF at 200 ng/mL resulted in an 18% escalation in the inflammatory reaction in contrast to the control. Remarkably, after administering diverse forms and dosages of PEA, all PEA variants notably subdued the inflammatory response compared to the induced damage. Notably, across all the concentrations examined, PEAΩ and PEADynoΩ exhibited a superior anti-inflammatory effect in contrast to all other formulations (*p* < 0.05); additionally, doses of 300 mg, 600 mg and 1200 mg demonstrated efficacy when compared to the remaining two doses tested (*p* < 0.05, 200 mg and 1800 mg). These results align with prior research indicating that among the PEA formulations evaluated, PEAΩ and PEADynoΩ displayed optimal outcomes at concentrations of 300 mg, 600 mg and 1200 mg. An anti-inflammatory effect directly correlated with dose may be shown by analysing indicators of inflammation, particularly in the 300–1200 mg range. Among all the variations of PEA, PEAΩ and PEADynoΩ were particularly remarkable at these levels, illustrating a significant reduction in inflammation. This decrease reached around 4.5-fold and 1.75-fold with a 300 mg dose, 4-fold% with a 600 mg dose and 4-fold% and 1.8-fold% with a 1200 mg dose compared to the commercial product. The results from the 1800 mg dosage were similar to those from the 1200 mg dosage; however, the data indicate that the impact tends to decrease after the 1200 mg level.

**Figure 6:**
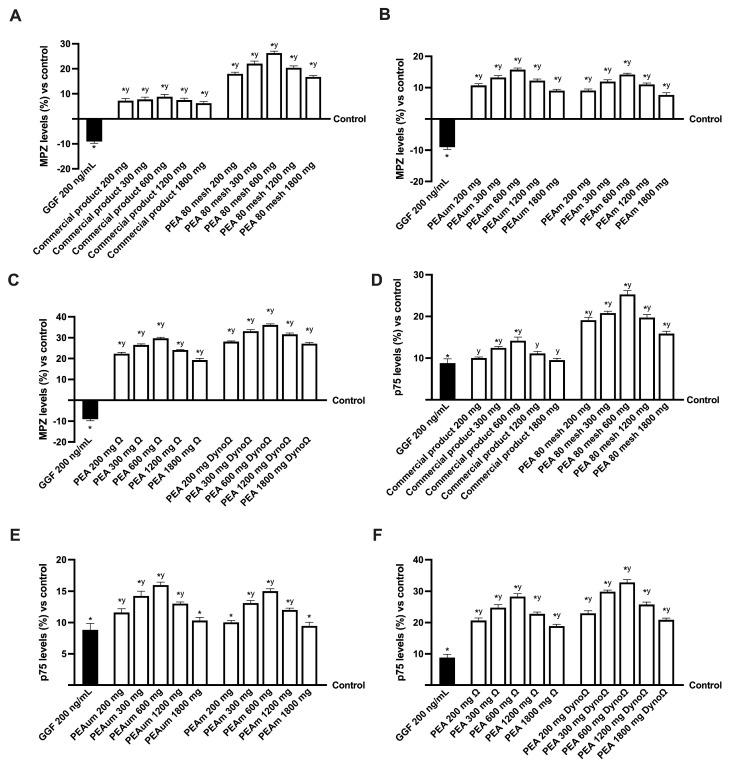
Analysis of the PEA formulations effects under PNI condition. (**A**–**C**) MPZ levels after treatment with all PEA forms at the concentration of different PEA forms; (**D**–**F**) p75 level after treatment with all PEA forms at the concentration of different PEA forms. The abbreviations are the same as those reported in Figure 4. Data are mean ± SD of five independent experiments performed in triplicates vs. control values (0% line, n = 5 independent observation per treatment group and each sample was run in triplicate). * *p* < 0.05 vs. control; ^y^ *p* < 0.05 vs. GGF 200 ng/mL.

Subsequently, the modulation of neuropathic pain was additionally investigated in an in vitro model. As reported in Figure 6, after nerve injury induced by 200 ng/mL GGF, myelinating cells were subjected to degradation as demonstrated by p75 analysis and, consequently, to the inhibition of myelin protein zero (MPZ) levels (about 8.83% and 9.1%, respectively, *p* < 0.05). In contrast, it was observed that all varieties and concentrations of PEA demonstrated the capability to mitigate harm, thereby validating their advantageous function in mitigating the process of demyelination (all indicators exhibited statistical significance of *p* < 0.05 in contrast to the damage). Particularly noteworthy is the superior efficacy of PEAΩ and PEADynoΩ compared to the other PEA formulations examined, as evidenced by their capacity to reverse the damage through the modulation of convoluted markers to uphold the customary operations of the protective sheath. When compared to the commercial product, PEAΩ and PEADynoΩ increased MPZ levels by approximately 7.8-fold and 3.3-fold at the 300 mg dosage, approximately 2.4-fold and 3-fold% at the 600 mg dosage and approximately 2.2-fold and 3.2-fold% at the 1200 mg dosage. Regarding p75 levels compared to the commercial product, PEAΩ and PEADynoΩ increased by approximately 98% and 1.4-fold at the 300 mg dosage, approximately 1-fold and 1.34-fold% at the 600 mg dosage and approximately 1.1-fold and 1.35-fold% at the 1200 mg dosage. In conclusion, 300 mg, 600 mg and 1200 mg were identified as providing the most substantial positive impact. According to data acquired on the MPZ and p75 markers, samples containing 1200 mg had positive effects like those of the relevant ones but at a lower dose of 300 mg, in the 300–1200 mg dosage range. The effectiveness of PEA has been reported to decrease with sample amounts greater than 1200 mg.

**Figure 7:**
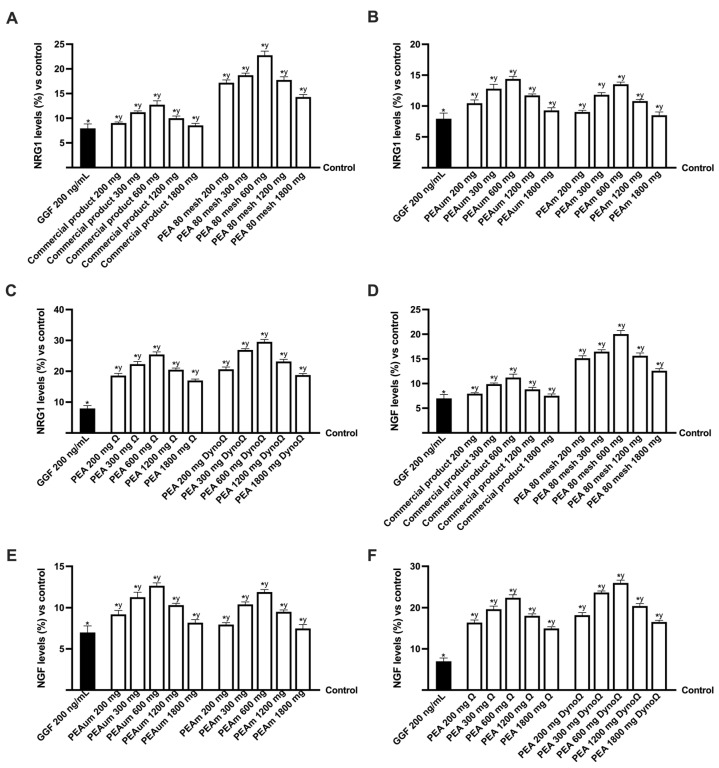
Analysis of the PEA formulation effects under PNI conditions. (**A**–**C**) NRG1 levels after treatment with all PEA forms are at the concentration of different PEA forms; (**D**–**F**) NGF levels after treatment with all PEA forms are at the concentration of different PEA forms. The abbreviations are the same as those reported in Figure 4. Data are mean ± SD of five independent experiments performed in triplicates vs. control values (0% line, n = 5 independent observation per treatment group and each sample was run in triplicate). * *p* < 0.05 vs. control; ^y^ *p* < 0.05 vs. GGF 200 ng/mL.

Finally, to validate the data acquired thus far, an examination was conducted on two additional indicators of peripheral regeneration, namely neuregulin 1 (NRG1) and nerve growth factor (NGF) (Figure 7). The findings indicated a decrease in the levels of these markers due to the damage caused by GGF 200 ng/mL; however, they were promptly reinstated by the positive impact of various forms and concentrations of PEA. This observation supports that both indicators enhance nerve damage recovery and promote the myelination process. Specifically, PEAΩ and PEADynoΩ demonstrated that their beneficial influence surpasses all other PEA variations tested. Furthermore, even when considering these two markers of peripheral nerve regeneration, the concentrations that exhibited superior outcomes were 300 mg, 600 mg and 1200 mg (*p* < 0.05). All of these findings indicate that the novel forms of PEAΩ and PEADynoΩ have the capability to effectively regulate the biological function of Schwann cells, even in the context of PNI. The outcomes showcased the significant impact of PEAΩ and PEADynoΩ in regulating Schwann cells throughout the progression and mitigation of nerve damage, implying a superior beneficial influence compared to existing treatments in the market. Depending on the NRG1 and NGF marker data, samples containing 1200 mg have been demonstrated to have positive effects comparable to those of their 300 mg equivalents within the 300–1200 mg dose range. When compared to the commercial product, PEAΩ and PEADynoΩ increased NRG1 levels by approximately 99% and 1.4-fold at the 300 mg dosage, approximately 1-fold and 1.33-fold at the 600 mg dosage and approximately 1.05-fold and 1.3-fold at the 1200 mg dosage. Regarding NGF levels compared to the commercial product, PEAΩ and PEADynoΩ increased by approximately 1-fold and 1.4-fold at the 300 mg dosage, approximately 1-fold and 1.32-fold% at the 600 mg dosage and approximately 1.1-fold and 1.32-fold% at the 1200 mg dosage. The effectiveness of PEA has been demonstrated to decrease with sample amounts greater than 1200 mg.

**Figure 8:**
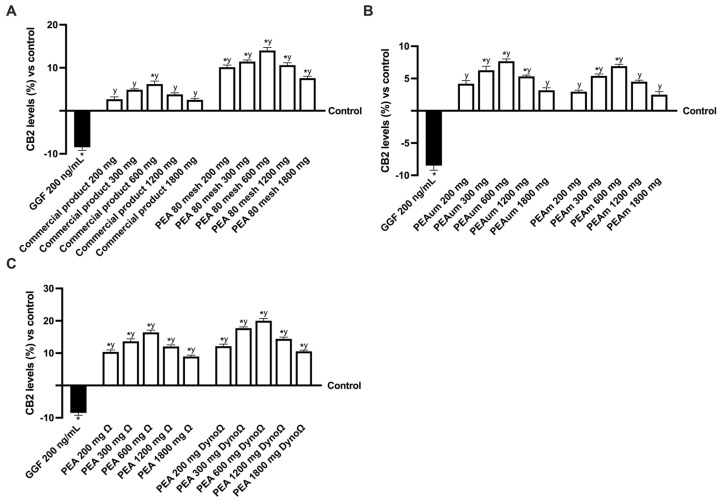
Analysis of CB2 levels after treatment of PEA formulation effects under PNI condition. In (**A**) CB2 analysis of Commercial product and PEA 80 mesh; in (**B**) CB2 analysis of PEA um and PEAm; in (**C**) CB2 analysis of PEA Ω and PEA DynoΩ. The abbreviations are the same as those reported in Figure 4. Data are mean ± SD of five independent experiments performed in triplicates vs. control values (0% line, n = 5 independent observation per treatment group and each sample was run in triplicate). * *p* < 0.05 vs. control; ^y^ *p* < 0.05 vs. GGF 200 ng/mL.

In addition, since the main target of PEA is the endocannabinoid system, the biological mechanisms of CB2 receptors and their role in the modulation of pain and its analgesic effect have been evaluated. As can be seen in Figure 8, all forms of PEA have confirmed their role as activators and inductors of increasing levels of CB2 compared to the control (*p* < 0.05) and the damage induced by GGF (*p* < 0.05). Compared to the commercial product, all forms of PEA have increased levels of CB2, except PEAm and PEAum, which perform comparably to the commercial product at the same dosage. The best data were recorded by PEAΩ and PEADyno, which almost doubled for PEA and doubled for Dyno technology regarding the activation levels of CB2 compared to the commercial product and PEAm and PEAum. From these results, it can be seen that for all the samples examined, the greatest activating effect was promoted by the 600 mg dosage, while the 300 mg and 1200 mg dosages showed comparable effects. Compared to the commercial product, PEAΩ and PEADynoΩ increased CB2 levels by about 1.8-fold and 2.62-fold at the 300 mg dose, about 1.6-fold and 2.2-fold at the 600 mg dose and 2.2-fold% and 2.8-fold at the 1200 dose, respectively.

## 3. Discussion

PEA is an endogenous fatty acid amide that may be encountered in numerous food sources of animal and plant origins [16]. PEA pertains to the NAE category of biologically active endogenous lipids, which encompasses the endogenous cannabinoid receptor ligand (AEA) and satiety factor (oleoylethanolamide) [39]. Furthermore, this amide is produced within the organism, under specific circumstances, from membrane phospholipids. PEA demonstrates diverse functions owing to its capacity to associate with distinct nuclear receptors to execute a broad array of functions against chronic pain and inflammation using PPAR-α. More precisely, it functions via a pleiotropic receptor-like mechanism entailing a receptor complex consisting of both membrane receptors (GPR55, CB2) and nuclear receptors (PPAR-α) [40]. Despite its noteworthy characteristics, this compound encounters challenges concerning its bioavailability, posing a substantial issue for the medical practitioner and the individual seeking treatment. Despite its significant attributes, the molecule encounters challenges related to bioavailability, a critical concern for healthcare providers and patients alike. Researchers have investigated various strategies to enhance the absorption of PEA, encompassing techniques such as micronisation and ultra-micronisation, combination formulations with antioxidants and the utilisation of vehicles, including apolar solvents, to optimise its bioavailability [41,42,43]. The present study examined the kinetics of intestinal absorption of diverse forms of PEA, administered at varying doses, to elucidate potential disparities in kinetics associated with the different particle sizes of all PEA forms. As reported in the literature, PEA can effectively counteract neuroinflammation at a cellular level when administered in micronised (a particle size range of 2–10 µm) or ultra-micronised (a particle size range of 0.8–6 µm) forms. In contrast, native-state PEA (naïve PEA) exhibits a weaker biological effect due to its larger particle size (100 to 2000 µm), resulting in poor absorption and reduced distribution and bioavailability [44]. Furthermore, when mPEA and umPEA are combined with natural compounds in co-micronised or co-ultramicronised forms, such as the antioxidant polyhydrin (e.g., mPEAPol), they display synergistic effects and increased biological activity. Numerous publications have highlighted the effectiveness of these PEA formulations—micronised, ultra-micronised, co-micronised and co-ultramicronised—in treating chronic pain conditions of various causes from a clinical perspective [45]. PEAm and PEAum consist of a crystalline form with particle sizes between 100 and 700 μm [21], characterised by a high surface-to-volume ratio that allows better diffusion and distribution and higher biological efficacy than non-micronised PEA. In 2016, however, Gabrielsson et al. suggested a cautious interpretation of the available literature on PEA due to conflicts of interest and the poor quality of some clinical trials [46]. Before discussing PEA’s efficacy in treating chronic pain, a crucial formulation question has to be answered. Since the absorption rate is inversely correlated with particle size, PEA’s tendency to aggregate into big particles (up to 2000 microns) poses a serious problem for the pharmaceutical industry. Micronisation procedures, which reduce the particle size to 0.8 microns, greatly enhance solubility and bioavailability [45]. This ensures the safety of PEA taken orally while also improving its potency. For these reasons, the micronised and ultra-micronised forms are recommended. They are the most studied in clinical practice, where the oral route is preferred due to the convenience of administration [47]. On the other hand, intraperitoneal distribution is typically the simplest and most popular mode of administration in lab animals, leading to a quicker and more thorough absorption than with oral delivery [7]. 

Furthermore, an assessment was performed regarding how distinct doses could be modulated differently at the intestinal level. Additionally, to assess the ultimate impact, the study aimed to evaluate the biological efficacy of all PEA forms at the peripheral nerve site under damaged conditions. This evaluation was crucial to comprehending how diverse doses influenced the activation of nerve regeneration processes starting from its different preparations. It is crucial to remember that there are a number of synthetic methods for preparing PEA. The main drawback of these methods is the presence of residual impurities or by-products (such as metals, catalysts or reagents) that reduce the chemical purity of the finished product, pose a risk to patient safety and have a detrimental effect on crystallisation and the subsequent manufacturing process. On the other hand, a synthetic process for PEA that does not require solvents or catalysts has been reported [48]. Compared to PEA powders made using other chemical synthesis methods, this synthetic method produced a crystalline PEA powder with higher purity [21]. In addition to that, it is more important to consider lipophilicity. Indeed, PEA is highly insoluble in water and poorly soluble in several other aqueous solvents, as indicated by an octanol–water partition coefficient (log P) higher than 5 [49]. The partition coefficient represents the ratio of unionised PEA distributed between the organic phase (octanol) and aqueous phases (water) at equilibrium. Accordingly, unprocessed PEA is ~100.000-fold more soluble in octanol than water. In addition, it is commonly dissolved in ethanol or dimethyl sulfoxide (DMSO), ranging from 0.2% to 1%, to make it suitable for addition to cell cultures without interfering with cell responses [50]. Regardless of its form, PEA is entirely non-toxic and harmless [22]. For this reason, the most carefully examined innovative PEA forms were PEAΩ and PEADynoΩ, developed using innovative technologies that enhanced their absorption kinetics without compromising their intrinsic biological characteristics upon reaching the target site. To test our hypothesis, we studied the absorption kinetics of different forms and concentrations of PEA using an in vitro model that simulates oral administration. Our findings revealed that all forms of PEA are safe and displayed absorption kinetics with a peak at 3 h of treatment, except PEADynoΩ, which showed a peak absorption time of 4 h for all concentrations tested. Additionally, our results showed that none of the forms and concentrations of PEA caused any damage to the cell monolayer, as they effectively maintained high TEER and TJ levels necessary for cell monolayer formation and integrity.

Furthermore, our findings revealed that none of the forms or concentrations of PEA damaged the cell monolayer, as they effectively maintained the high levels of TEER and TJ required for the cell monolayer’s development and stability. One important issue with PEA is its low bioavailability when delivered naturally, often known as naïve PEA. Native PEA has a large particle size of 100 to 2000 microns, which causes low solubility and restricted absorption in the gastrointestinal system. These limitations reduce its effectiveness in achieving therapeutic levels in the body [29]. Micronisation has decreased particle size in the micron or submicron range. PEAm generally has particle sizes ranging from 2 to 10 microns, whereas PEAum is between 0.8 and 6 microns. These smaller particles have a larger surface-to-volume ratio, which improves their solubility and, hence, their bioavailability [46]. Studies indicate that PEAm is more effective than naïve PEA in treating chronic pain and neuroinflammation [7]. PEA’s effectiveness is also dependent on its dosage. Clinical studies indicate that PEAm and PEAum are more efficacious at lower dosages than naïve PEA due to increased bioavailability. PEAum can provide considerable pain relief at dosages as low as 300 mg/day, but naïve PEA requires greater doses to elicit equal benefits, leading to variable therapeutic results [14]. Based on these findings, PEA’s production process and dose are critical in determining its therapeutic efficacy. 

The gut-level analyses allowed us to determine an appropriate dosage range for each sample, ranging from 300–1200 mg. Furthermore, in vitro intestinal barrier integrity studies revealed a similarity of effects between the 300 and 1200 mg dosages, with a decline in the effects of PEA in the various forms and technologies tested exceeding the 1200 mg level. On the other hand, regarding targeting, by simulating the neuropathy condition in vitro, specifically inflammation and oxidative stress leading to an imbalance in ion transport, the data revealed that the investigational substances at all dosages tested seem to be able to repair the damage to the myelin sheath that protects the axon and simultaneously act on NGF release and bind to the neurotrophin p75 receptor, reproducing the mechanism of analgesia observed in humans. Indeed, the state of oxidant/antioxidant equilibrium has a significant impact on the viability of cells as revealed by PEA stimulations; PEA has been demonstrated to lower ROS levels by increasing the activity of endogenous antioxidant systems, preserving the integrity and viability of cells and function, which may delay the course of the damage. These biological processes have significant therapeutic ramifications, especially for the treatment of inflammation, chronic pain and neurodegenerative illnesses [51]. Indeed, as reported in the literature [51], an increase in NGF levels in various inflammatory conditions can be considered an important hallmark of the human chronic pain condition. PPAR-α is the main receptor through which PEA operates. Still, it modifies other pain-related receptors, including cannabinoid receptors CB1 and CB2, TRPV1 and others [52,53]. PEA has been demonstrated to decrease inflammation and neuroinflammation in cellular models of neuropathic pain by lowering microglial activation and regulating the production of pro-inflammatory cytokines, including TNF-α and IL-1β [4,54]. The ability of PEA to prevent mast cell degranulation—an immune cell involved in the inflammatory response and neural sensitisation—has also been validated by more recent in vitro investigations. This effect is critical in continuing pain in neuropathic pain, where peripheral and central sensitisation are important components [17,55]. Therefore, a reduction in the inflammatory response was important to counteract the negative consequences of chronic pain; in fact, our study showed that all the substances and concentrations tested could reduce the inflammatory response after damage induction with GGF 200 ng/mL. In contrast, pain is linked to a functional imbalance between MPZ and NRG1, which is crucial in maintaining Schwann cell homeostasis during PNI. NRG1 normally activates ERB receptors in peripheral nerves to regulate various functions of Schwann cells, such as growth, migration, differentiation and dedifferentiation. However, PNI disrupts NRG1/ERb signalling by affecting the balance of NRG1 isoforms and reducing the expression of molecules involved in cell survival, activating the MAPK pathway [56]. The sensory neurodegeneration observed in PNI is associated with impaired neurotrophic support and disruption of NRG-1/ERb signalling, potentially affecting the biological activity of Schwann cells. Nevertheless, treatment with the studied substances has been shown to restore impaired neurotropism, preventing the slowing of nerve conduction and damage to motor neurons. It is well-known that cells respond to nerve damage by changing their characteristics, proliferating and interacting with nociceptive neurons by releasing glial mediators (growth factors, cytokines, chemokines and biologically active small molecules) [57]. Furthermore, the receptors expressed in activated Schwann cells have the potential to regulate their communication with axons, thereby facilitating the regeneration of the myelin sheath and protecting the nerve from further harm [58]. Our results at the nerve target level confirmed the optimum and therapeutic role of the 300–1200 mg dose range. Analyses on markers of well-being and peripheral nerve function such as MPZ, NGR1, p75 and NGF allowed us to determine that the various forms of PEA at 600 mg had a stronger effect than the other PEAΩ samples and the commercial product alone. The data reported for the 1200 mg dosage were equivalent to the 300 mg dosage; over 1200 mg, the efficacy of PEA at the nerve level tends to decline. Furthermore, because the role of PEA in interacting with CB2 has been identified, PEA has an important analgesic role in pain control. It is a chemical that has no known negative effects when consumed in the recommended amounts [59]. Adults typically receive a daily dose of 1200–1600 mg. Our study found that PEA has a stronger stimulatory effect on CB2 levels at the nerve level than commercial products, PEAum, PEAm and PEA combined with Simbio and Dyno technologies. The 300 mg to 1200 mg dosage range produced excellent results, particularly the 600 mg dose in which PEAΩ and PEA DynoΩ increased CB2 levels by 16% and 20% compared to the commercial products PEAum and PEAm at 6–8%. The 300 mg and 1200 mg dosages produced similar results, with a decrease in PEA efficacy above 1200 mg. The results highlight the impact of PEA’s particle size and concentration adjustments on the modification of its kinetics, consequently affecting the specific biological effects exerted by PEA on the end target in the context of PNI.

## 4. Materials and Methods

### 4.1. Agents Preparation

PEA in several forms, micronised (m), ultra-micronised (um) and 80 mesh, were prepared in Dulbecco’s Modified Eagle’s Medium without red phenol (DMEM, GIBCO^®^ ThermoFisher Scientific, Waltham, MA, USA) and fetal bovine serum (FBS, Merck Life Science, Rome, Italy), 2 mM L-glutamine and 1% penicillin-streptomycin (Merck Life Science, Rome, Italy) and 1 mM sodium pyruvate (Merck Life Science, Rome, Italy) to stimulate the intestinal cells. In addition, PEA 80 mesh was also prepared in Ω (technology based on solvent patent N°102017000036744) and DynoΩ (technology based on solvent patent N° 102024000003076). All PEA forms were prepared directly on the medium, based on their specific solubility: PEAm and PEAum had a reduction in particle size, leading to improved distribution in water-based solvents; PEA 80 mesh using sub-micronisation/granulation had slight solubility in water-based solvents and PEAΩ and DynoΩ contained solubilising agents. The effects were compared to those of the commercial product (mainly composed of UM form), used at the same concentrations and conditions as the other PEA forms to ensure a positive control. The concentration of each PEA form was chosen from the literature [60], which refers to the human dose, starting from a preparation diluted to 1:2000 as a coefficient to reflect human physiological conditions [61]. Successively, the experiments examined the impacts following the passage through the intestines of various substances on the peripheral nerve injury caused by 200 ng/mL GGF (Tebu-Bio, Magenta, Milan, Italy), a widely accepted model for simulating PNI due to its ability to replicate significant demyelination [62]. Moreover, 200 ng/mL GGF was introduced directly into the medium within the 3D EngNT to trigger demyelination.

### 4.2. Cell Culture

The human epithelial intestinal cell line, CaCo-2, obtained from the American Type Culture Collection (ATCC, Manassas, VA, USA), was employed as a model of the intestinal barrier based on the characteristics described in the literature regarding its ability to predict the intestinal absorption of substances in humans [63]. This cell line was cultured in Advanced Dulbecco’s Modified Eagle’s Medium/Nutrient F-12 Ham (Adv DMEM-F12; GIBCO^®^ ThermoFisher Scientific, Waltham, MA, USA) containing 10% fetal bovine serum (FBS, Merck Life Science, Rome, Italy), 2 mM L-glutamine and 1% penicillin-streptomycin (Merck Life Science, Rome, Italy) and maintained in an incubator at 37 °C and 5% CO_2_ [37]. Experiments employed cells with passage numbers ranging from 26 to 32 to preserve the appropriate paracellular permeability and transport characteristics [64]. Furthermore, 1.8 × 10^4^ cells were seeded onto a 6.5 mm Transwell^®^ insert (Corning^®^ Costar^®^, Merck Life Science, Rome, Italy) with a 0.4 μm pore size polycarbonate membrane (Corning^®^ Costar^®^, Merck Life Science, Rome, Italy) in a 24-well plate for conducting absorption studies [65]. The Transwell^®^ insert-plated cells were kept in a complete medium for 21 days to reach maturation, with changes made every other day on both the basolateral and apical sides. The medium was brought to pH 6.5, representing the small intestine’s lumen on the apical side before stimulation. In contrast, pH 7.4, which represents blood, was placed on the basolateral side [66]. 

The human intestinal mucus-secreting cell line HT29-MTX was generously provided by Professor Isidoro’s Molecular Pathology and NanoBioImaging (Univesity of Piemonte Orientale, UPO, Novara, Italy). Cells were cultivated in Adv-DMEM with 5% FBS, 2 mM L-glutamine and 1% penicillin-streptomycin, and kept at 37 °C in an incubator with 5% CO_2_. To maintain the proper mucus-secreting phenotypes, the cells used in the tests were at passages between 10 and 20 [67]. Specifically, to investigate intestinal permeability, 2 × 10^3^ cells were loaded onto a 6.5 mm Transwell^®^ with a 0.4 μm pore polycarbonate membrane insert (Merck Life Science, Rome, Italy) in 24-well plates. Before the treatment, the cells were plated on a Transwell^®^ insert and kept in a complete medium that was replaced every other day, first basolaterally and then apically; the protocol was followed for 28 days until maturation of the cells [68]. 

RSC-96 cells, a Schwann cell line derived from rats, were procured from ATCC and grown in Adv DMEM (GIBCO^®^ ThermoFisher Scientific, Waltham, MA, USA) supplemented with 5% FBS, 2 mM L-glutamine and 1% penicillin–streptomycin [69]. The cell cultures were maintained at 37 °C with 5% CO_2_. The subculturing of RSC96 cells was performed 2–3 times per week, with passages ranging between 10 and 15 utilised for the experimental procedures [70].

The rat neuronal PC12 cell line, supplied by ATCC, was cultured in Roswell Park Memorial Institute-1640 (RPMI, Merck Life Science, Rome, Italy) supplemented with 2 mM glutamine, 10% horse serum (HS; Merck Life Science, Rome, Italy) and 5% FBS. The cultures were carefully maintained at sub-confluency in an incubator set at 37 °C with 5% CO_2_ and 95% humidity. The cells utilised for experiments had undergone between 3 and 13 passages [71]. The concentration of 4 × 10^6^ RSC96 cells and 1 × 10^5^ PC12 cells was the most suitable for seeding a co-culture system to replicate the 3D EngNT in the peripheral nerve environment [69].

### 4.3. Experimental Protocol

**Figure 9:**
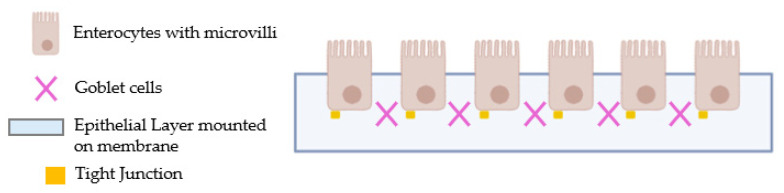
Schematic illustration of the intestinal in vitro model.

The studies were divided into two phases: the first one examined the absorption effects of all PEA formulations on CaCo-2/HT29-MTX co-cultures (Figure 9) through a dose–response in vitro intestinal model to evaluate their intestinal passage across the intestinal barrier through a fluorescent probe and the barrier’s integrity using TEER. Indeed, the PEA concentrations selected were also used in an in vitro intestinal permeability assay to evaluate the ability to cross the intestinal barrier and maintain appropriate TJ activity, confirming intestinal integrity via TEER assessment. All these experiments on the absorption were time-dependent, ranging from 0.5 to 6 h, while TJ levels were evaluated after 6 h of treatment [72]. At the end of each simulation, the basolateral medium was also collected to stimulate the 3D peripheral nerve model. 

**Figure 10:**
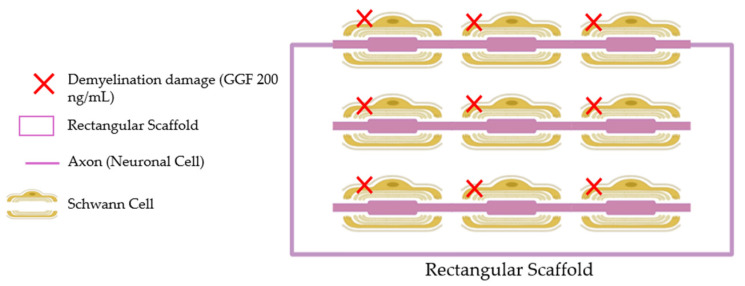
Schematic illustration of the 3D EngNT co-culture in vitro model.

In the second phase of the protocol, the 3D EngNT co-culture was chosen to investigate the effect of the stimulation with PEA samples on the nerve tissue model in vitro; before usage, the prototype reached maturity in 14 days, and the stimulation time was 24 h. In particular, the 3D EngNT underwent pre-treatment commencing on day 14 of maturation with a concentration of 200 ng/mL GGF to replicate substantial demyelination before exposure to various forms of PEA (Figure 10). Within this context, an assessment was conducted on cell viability, ROS production, TNFα, NRG1, p75 neurotrophin receptor, MPZ, NGF and CB2 levels utilising specific assay kits.

### 4.4. Cell Viability

Cell viability based on the In Vitro Toxicology Assay Kit (Merck Life Science, Rome, Italy) was assessed at the end of each stimulation, following a classical protocol reported in the literature [73]. Using a spectrometer (Infinite 200 Pro MPlex, Tecan, Männedorf, Switzerland), the absorbance of each solubilised sample was measured at 570 nm and corrected at 650 nm. As the means of five separate experiments carried out in triplicate, the results were reported and expressed by comparison with the control sample, defined as untreated samples and represented by the 0% line.

### 4.5. ROS Production and Measurement

Superoxide anion release was quantified using a standard methodology based on cytochrome C reduction [74]. The absorbance in the culture supernatants was measured at 550 nm using a Tecan spectrophotometer after adding 100 μL of cytochrome C (Merck Life Science, Rome, Italy) to each well. Comparatively, the plate was incubated for 30 min after empty wells were filled with 100 μL of cytochrome C and 100 μL of superoxide dismutase (Merck Life Science, Rome, Italy). Compared to the control (0 line), the O_2_ rate was measured as the average standard deviation (%) of nanomoles per reduced cytochrome C per microgram of protein.

### 4.6. In Vitro Intestinal Barrier Model

The CaCo-2 cells (enterocytes) were placed in a co-culture with HT29-MTX cells (goblet cells) in a 9:1 ratio (Caco-2:HT29-MTX) [75]. Throughout the development period, the TEER values were assessed using EVOM3 in conjunction with STX2 chopstick electrodes (World Precision Instruments, Sarasota, FL, USA) to assess the creation of mature intestinal epithelium and an appropriate paracellular mechanism. On the 21st day, when TEER values were ≥495 Ω*cm^2^ [75], absorption analysis had started. Before the stimulation, on the apical side, the medium was brought to pH 6.5, the pH in the lumen of the small intestine, while pH 7.4 on the basolateral side represented blood [66]. The cells were stimulated with all substances from 0.5 h to 6 h before successive analyses, including the permeation rate [nmol min (mg protein)] [76], which follows the following formula:

J = Jmax [C]/(Kt + [C])(1)

where: C: the initial concentration of fluorescein. 

Jmax: the maximum permeation rate. 

Kt: the Michaelis–Menten constant. 

The results are expressed as the means ± SD (%). Negative controls without cells were tested to exclude the Transwell^®^ membrane’s influence. The analysis was performed in five separate experiments carried out in triplicate.

### 4.7. Claudin-1 ELISA Assay

Human Claudin-1 was measured in CaCo-2/HT29-MTX co-culture lysates using an ELISA kit (Cusabio Technology LLC, Houston, TX, USA) in accordance with the manufacturer’s instructions [77]. After lysing cells in cold PBS (Merck Life Science, Rome, Italy) at a ratio of 1×, the samples were centrifuged at 1500× *g* for 20 min at 4 °C. A spectrometer (Infinite 200 Pro MPlex, Tecan, Männedorf, Switzerland) examined and read 100 μL of each sample. Five independent experiments were conducted in triplicate, and the results were expressed as a mean ± SD (%) vs. the control (0 line) after comparing the data to the standard curve, which spans from 0 to 1000 pg/mL.

### 4.8. Occludin ELISA Assay

The level of Occludin was measured using the Human Occludin (OCLN) ELISA Kit (MyBiosource, San Diego, CA, USA) following the manufacturer’s instructions [72]. After lysing CaCo-2/HT29-MTX co-culture in cold Phosphate-Buffered Saline (PBS, Merck Life Science, Rome, Italy) 1×, each sample was subjected to 20 min centrifugation at 1500× *g* at 4 °C, and 100 μL was analysed. A spectrometer (Infinite 200 Pro MPlex, Tecan, Männedorf, Switzerland) was used to analyse the enzymatic reaction at 450 nm. The data were compared to the standard curve, which spans 0 to 1500 pg/mL, to produce the results, which were then expressed as a percentage (%) against the control (0 line) of five separate experiments carried out in triplicate.

### 4.9. Human Tight Junction Protein 1 (ZO-1) ELISA Assay

The manufacturer’s instructions for the human tight junction protein 1 (TJP1) ELISA kit (MyBiosource, San Diego, CA, USA) were followed [78]. A volume of 100 μL was evaluated for each sample after the CaCo-2/HT29-MTX co-culture was lysed with cold PBS 1× (Merck Life Science, Rome, Italy), centrifuged at 5000× *g* for 20 min at 4 °C and read at 450 nm with a spectrometer (Tecan Infinite 200 Pro MPlex, Männedorf, Switzerland). The standard curve, which spans from 0 to 1000 pg/mL, was compared with the collected data, and the outcomes were reported as the mean ± SD (%) of five separate tests conducted in triplicate, as opposed to the control (0 line).

### 4.10. 3D EngNT Co-Cultures Setup

According to the literature, the 3D nerve tissue model was prepared [37]. The interaction between RSC96 and PC12 cell lines is a key feature for mimicking the in vitro peripheral nerve environment, regenerating neurites and supporting Schwann cells [37,79]. Specifically, 1 mL of solution was added to a rectangular scaffold with 16.4 mm × 6.5 mm × 5 mm dimensions. The solution contained 80% *v*/*v* Type I rat tail collagen (2 mg/mL in 0.6% acetic acid from Thermo Fischer, Milan, Italy), 10% *v*/*v* Minimum Essential Medium (MEM from Merck Life Science, Rome, Italy), 5.8% *v*/*v* neutralising solution (from Biosystems, Monza, Italy) and 4.2% Schwann cell suspension (4 × 10^6^ RSC96 cells per 1 mL gel). After the gel had been set, it was placed in 10 mL of DMEM and incubated at 37 °C with 5% CO_2_ for 24 h to allow for cellular self-alignment. The gel was then stabilised by applying plastic compression (120 g weight for 1 min). Once the gel had been properly aligned and stabilised, it was divided into equal segments based on the samples that needed to be treated. Each segment of the gel was carefully transferred to a 24-well plate. Next, 1 × 10^5^ PC12 cells were placed on the top of each segment to establish the co-cultures. This step is essential as it allows for the extension of neurites across the gel. The 24-well plate containing the gels was then incubated at 37 °C for 1 h, allowing the neuronal cells to attach to the collagen gel. Finally, 1 mL of the culture medium (consisting of DMEM supplemented with 10% FBS, 100 U/mL of Penicillin and 100 μg/mL of Streptomycin purchased from Merck Life Science, Rome, Italy) was added to each well. Before the stimulation, the 3D nerve tissue model was pre-treated with 200 ng/mL GGF after 14 days of maturation until substantial demyelination was achieved.

### 4.11. TNF-α ELISA Assay

TNFα levels were measured using the TNF-α ELISA kit (Merck Life Science, Milano, Italy) following the instructions provided by the manufacturer [70]. The absorbance of the samples was recorded at 450 nm using a plate reader (Infinite 200 Pro MPlex, Tecan, Männedorf, Switzerland). The results were presented as mean ± SD (%) compared to the control (line 0) in five separate experiments conducted in triplicate.

### 4.12. NRG1 ELISA Assay

The NRG1 Rat ELISA Kit (FineTest, Wuhan, China) was used in EngNT supernatants, following the instructions provided by the manufacturer [18]. In each well, 100 µL of each sample was added, and the plate was then incubated at 37 °C for 90 min. After the incubation, each well’s material was removed, and the wells were washed twice with a Wash Buffer. Next, 100 µL of the biotin-labelled antibody working solution was added to the wells and the plate was incubated at 37 °C for 60 min. Following incubation, the solution in each well was removed, and the wells were washed three times with a Wash Buffer. Subsequently, 100 µL of the SABC Working Solution was added to each well, and the plate was incubated at 37 °C for 30 min. In the end, the wells were washed five times, and 90 µL of the TMB substrate was added to each well. After 10–20 min, 50 µL of the Stop Solution was added to each well, and the plate was immediately read at 450 nm using a plate reader (Infinite 200 Pro MPlex, Tecan, Männedorf, Switzerland). The data were obtained and compared to the standard curve (ranging from 0.156 to 10 ng/mL), and the results were expressed as means ± SD (%) vs. control (0 line) of five independent experiments performed in triplicate.

### 4.13. NGFR ELISA Assay

The Rat NGFR ELISA kit (MyBioSource, San Diego, CA, USA) was utilised on 3D EngNT lysates in accordance with the manufacturer’s instructions [80]. Using a spectrophotometer (Infinite 200 ProMPlex, Tecan, Männedorf, Switzerland), the plate was read at 450 nm. The acquired data were compared to the standard curve, which ranges from 0.312 to 20 ng/mL. The findings were presented as the mean ± SD (%) of five separate triplicate tests compared to the control (0 lines).

### 4.14. MPZ ELISA Kit

Using a Rat ELISA kit (MyBiosource, San Diego, CA, USA) for 3D EngNT lysates, the synthesis of myelin protein zero (MPZ) was measured in accordance with the manufacturer’s instructions [37]. In summary, 100 µL of 3D EngNT lysates was carefully added to each well, and the plate was incubated at 37 °C for a duration of two hours. Upon completion of all reactions, 50 µL of Stop Solution was gently applied to each well, and the plate was promptly measured at 450 nm using a Tecan Infinite 200 Pro MPlex spectrometer. The concentration was determined by comparing it to a standard curve ranging from 0.06 to 18 ng/mL. The results were presented as means ± SD (%) compared to the control (line 0) in five separate experiments conducted in triplicate.

### 4.15. Human Beta-NGF Assay

The Human beta-NGF ELISA kit (Abcam, Cambridge, UK) was utilised in cell lysates according to the manufacturer’s instructions as reported in the literature [81]. In summary, 100 μL of diluted samples were incubated overnight at 4 °C, followed by four washes with 1× Wash Buffer. Subsequently, 100 μL of the detection antibody was added to each well and incubated for 1 h at room temperature with gentle shaking. The wells were then washed four times, and 100 μL of streptavidin-HRP was added to the plate and then incubated for 45 min. After the incubation, the wells were washed again, and 100 μL of the TMB substrate was added. Finally, the plate was incubated for 30 min at room temperature in the dark with gentle shaking, and the reaction was halted with 50 μL of Stop Solution. The absorbance was measured by the spectrometer at 450 nm (Infinite 200 Pro MPlex, Tecan, Männedorf, Switzerland). The results were expressed as pg/mL compared to a standard curve (ranging from 15 to 15,000 pg/mL), with the findings reported as the mean ± SD (%) compared to the control from five independent experiments conducted in triplicate.

### 4.16. CB2 ELISA Kit

The CNR2 ELISA Kit (FineTest, Wuhan, China) was used in EngNT supernatants, following the instructions provided by the manufacturer to verify the function of the CB2 receptor [82]. In each well, 100 µL of each sample was added, and the plate was then incubated at 37 °C for 90 min. After the incubation, each well’s material was removed, and the wells were washed twice with a Wash Buffer. Next, 100 µL of the biotin-labelled antibody working solution was added to the wells and the plate was incubated at 37 °C for 60 min. Following incubation, the solution in each well was removed, and the wells were washed three times with a Wash Buffer. Subsequently, 100 µL of the SABC Working Solution was added to each well, and the plate was incubated at 37 °C for 30 min. In the end, the wells were washed five times, and 90 µL of the TMB substrate was added to each well. After 10–20 min, 50 µL of the Stop Solution was added to each well, and the plate was immediately read at 450 nm using a plate reader (Infinite 200 Pro MPlex, Tecan, Männedorf, Switzerland). The data were obtained and compared to the standard curve (ranging from 62.5 to 4000 pg/mL), and the results were expressed as mean ± SD (%) vs. control (0 line) of five independent experiments performed in triplicate.

### 4.17. Statistical Analysis

For datasets undergoing statistical analysis, we have established a minimum “n” of 5 independent observations per group. Each sample was run three times (“in triplicate”) using the pseudoreplication to test the reliability of the single values obtained [83,84]. The data acquired using Prism GraphPad statistical software 9.4.1 were processed using one-way analysis of variance (ANOVA) and Bonferroni post hoc tests. Student’s t-test with two tails was adopted to compare the two groups. A two-way ANOVA was conducted to evaluate multiple group comparisons, followed by a two-sided Dunnett post hoc test. The mean ± SD of at least five independent variables reproduced in triplicates was used to express all results, commonly used as a measure of central tendency.

## 5. Conclusions

In conclusion, the present investigation assessed the impact of processes called ultra-micronisation, or the employment of apolar carriers, on the modulation of PEA uptake kinetics while preserving the advantageous characteristics of the compound. Moreover, our analysis brings to the forefront, as the initial instance, the varying modulation effects of different concentrations on the mechanisms of regeneration and recovery at the neural level in the damaged peripheral nerve. This enabled us to illuminate the observation that low or high concentrations of PEA may, at times, be ineffectual in biological reactivity, thereby indicating the necessity for heightened attention to the dosage of administration. The dose must be carefully tuned to balance efficacy and safety, and the method of administration should be selected based on the clinical situation. Furthermore, guaranteeing the purity and quality of PEA formulations is critical to attaining consistent therapeutic results. To fully utilise PEA to manage chronic pain and inflammation, it is important to consider these factors. As a result of our investigation, it was reasonable to identify a dose range of greater efficacy at the level of the nervous target for all types of PEA investigated, which was identified as the range of 300–600 mg. Moreover, future research should use a more complex system to test physiologically realistic concentrations of PEA’s forms to confirm these observations. 

## Figures and Tables

**Table 1:** Cell viability of 3D EngNT in peripheral nerve injury (PNI) conditions. GGF = glial growth factor 2; the other abbreviations are the same as those reported in Figure 1. Data are mean ± SD of five independent experiments performed in triplicates vs. control values (0% value, n = 5 independent observation per treatment group and each sample was run in triplicate). α *p* < 0.05 vs. Commercial product; β *p* < 0.05 vs. PEAm; γ *p* < 0.05 vs. PEAum; φ *p* < 0.05 vs. GGF 200 ng/mL.

Sample	200 mg (%)	300 mg (%)	600 mg (%)	1200 mg (%)	1800 mg (%)
GGF 200 ng/mL	−8.50 ± 1.23	−8.50 ± 3.20	−8.50 ± 1.98	−8.50 ± 3.41	−8.50 ± 2.46
^φ^ Commercial product	0.98 ± 0.12	13.69 ± 4.89	11.41 ± 7.89	9.67 ± 2.45	2.56 ± 1.45
^αφ^ PEAm	5.43 ± 2.13	20.54 ± 3.66	16.40 ± 5.77	12.56 ± 4.37	8.43 ± 4.51
^αφ^ PEAum	8.32 ± 1.56	21.76 ± 8.10	18.10 ± 6.43	16.37 ± 4.88	10.34 ± 4.95
^αβγφ^ PEA 80 mesh	15.87 ± 4.56	27.71 ± 10.2	31.79 ± 11.4	29.91 ± 13.2	20.78 ± 10.4
^αβγφ^ PEAΩ	24.44 ± 6.39	32.82 ± 10.5	37.22 ± 9.26	34.12 ± 3.17	28.11 ± 10.9
^αβγφ^ PEA DynoΩ	22.50 ± 8.75	30.34 ± 11.8	35.15 ± 5.64	32.21 ± 6.91	25.96 ± 7.24

## Data Availability

The Laboratory of Physiology (F.Uberti) collects raw data and takes appropriate procedures to preserve them in a secure system forever. The corresponding author can provide this study’s data upon reasonable request.

## Abbreviations

Adv DMEM-F12	Advanced Dulbecco’s Modified Eagle’s Medium/Nutrient F-12 Ham
AEA	anandamide
AEA	endocannabinoid anandamide
ALIA	autacoid local inflammatory antagonism
ANOVA	one-way analysis of variance
ATCC	American Type Culture Collection
CB1	cannabinoid receptors 1
CB2	cannabinoid receptors 2
COX	cyclooxygenase
DMEM	Dulbecco’s Modified Eagle’s Medium
FBS	foetal bovine serum
GGF	glial growth factor
GPR119	G protein-coupled receptor 119
GPR55	G-protein-coupled receptor 55
HS	horse serum
MEM	Minimum Essential Medium
MPZ	myelin protein zero
NAEs	N-acyl ethanolamines
NGF	nerve growth factor
NRG1	neuregulin 1
PEA	Palmitoylethanolamide
PEAm	PEA micronised
PEAum	PEA ultra-micronised
PEA 80 mesh	PEA with particle size around 80 mesh
PEAΩ	PEA with particle size around 80 mesh prepared in Ω
PEADynoΩ	PEA with particle size around 80 mesh prepared in DynoΩ
PNI	peripheral nerve injuries
PPAR-α	receptor alpha peroxisome proliferator-activated receptor
ROS	reactive oxygen species
RPMI	Roswell Park Memorial Institute-1640
TEER	trans epithelial electrical resistance
TJ	tight junction
TNFα	tumour necrosis factor α
TRPV1	transient receptor potential vanilloid receptor 1

## Appendix B

**Table A1:** Time-course (dose 200 mg) studies of intestinal barrier integrity on Transwell^®^ by TEER measurements. α *p* < 0.05 vs. Commercial product; β *p* < 0.05 vs. PEAm; γ *p* < 0.05 vs. PEAum; δ *p* < 0.05 vs. PEA 80 mesh.

	Sample	Commercial Product 200 mg	PEAm 200 mg	PEAum 200 mg	PEA80mesch 200 mg	PEA 200 mg Ω	PEA 200 mg DynoΩ
Time							
0.5 h		428 ± 12.3	418 ± 6.25	^αβ^ 440 ± 21.3	^αβ^ 435 ± 14.8	^αβδ^ 452 ± 27.4	^αβδ^ 440 ± 15.8
1 h		438 ± 10.5	428 ± 13.6	^αβ^ 455 ± 19.7	^αβ^ 445 ± 18.7	^αβδ^ 459 ± 24.2	^αβδ^ 455 ± 21.2
2 h		448 ± 16.7	438 ± 14.9	^αβ^ 460 ± 22.1	^αβ^ 455 ± 20.4	^αβδ^ 462.5 ± 25.1	^αβδ^ 460 ± 24.5
3 h		458 ± 9.56	448 ± 19.3	^αβ^ 465 ± 25.4	^αβ^ 460 ± 24.6	^αβδ^ 470 ± 28.4	^αβδ^ 465 ± 23.1
4 h		440 ± 17.2	430 ± 18.1	^αβ^ 445 ± 16.4	^αβ^ 455 ± 13.7	^αβγδ^ 465 ± 13.7	^αβγδ^ 468 ± 22.7
5 h		420 ± 10.2	410 ± 5.23	^αβ^ 415 ± 9.63	^αβ^ 430 ± 10.6	^αβγδ^ 455 ± 11.4	^αβγδ^ 463 ± 20.4
6 h		414 ± 8.49	410 ± 4.26	^αβ^ 410 ± 5.75	^αβ^ 425 ± 11.9	^αβγδ^ 435 ± 10.9	^αβγδ^ 461 ± 19.6

**Table A2:** Time-course (dose 300 mg) studies of intestinal barrier integrity on Transwell^®^ by TEER measurements. α *p* < 0.05 vs. Commercial product; β *p* < 0.05 vs. PEAm; γ *p* < 0.05 vs. PEAum; δ *p* < 0.05 vs. PEA 80 mesh.

	Sample	Commercial Product 300 mg	PEAm 300 mg	PEAum 300 mg	PEA80mesch 300 mg	PEA 300 mg Ω	PEA 300 mg DynoΩ
Time							
0.5 h		^β^ 438 ± 11.0	428 ± 9.80	^αβ^ 450 ± 19.7	^αβ^ 445 ± 16.8	^αβγδ^ 462 ± 12.5	^αβδ^ 450 ± 13.8
1 h		^β^ 448 ± 12.8	438 ± 13.6	^αβ^ 465 ± 24.2	^αβ^ 455 ± 16.6	^αβδ^ 469 ± 17.9	^αβδ^ 465 ± 14.1
2 h		^β^ 458 ± 11.9	448 ± 22.3	^αβ^ 470 ± 23.5	^αβ^ 465 ± 15.3	^αβδ^ 472.5 ± 26.8	^αβδ^ 470 ± 14.5
3 h		^β^ 468 ± 114.4	458 ± 21.9	^αβ^ 475 ± 29.7	^αβ^ 470 ± 11.9	^αβγδ^ 480 ± 24.5	^αβδ^ 475 ± 13.6
4 h		^β^ 450 ± 12.1	440 ± 18.6	^αβ^ 455 ± 16.3	^αβ^ 465 ± 13.8	^αβγδ^ 475 ± 23.4	^αβγδ^ 478 ± 14.2
5 h		^β^ 430 ± 10.7	420 ± 15.8	^αβ^ 425 ± 14.1	^αβ^ 440 ± 17.1	^αβγδ^ 465 ± 19.9	^αβγδ^ 473 ± 15.8
6 h		^β^ 424 ± 11.4	420 ± 10.7	^αβ^ 420 ± 11.2	^αβ^ 435 ± 19.7	^αβγδ^ 445 ± 14.2	^αβγδ^ 471 ± 16.9

**Table A3:** Time-course (dose 600 mg) studies of intestinal barrier integrity on Transwell^®^ by TEER measurements. α *p* < 0.05 vs. Commercial product; β *p* < 0.05 vs. PEAm; γ *p* < 0.05 vs. PEAum; δ *p* < 0.05 vs. PEA 80 mesh.

	Sample	Commercial Product 600 mg	PEAm 600 mg	PEAum 600 mg	PEA80mesch 600 mg	PEA 600 mg Ω	PEA 600 mg DynoΩ
Time							
0.5 h		^β^ 460 ± 11.3	425 ± 12.4	^αβ^ 435 ± 10.5	^αβ^ 459 ± 12.9	^αβγδ^ 470 ± 18.5	^αβγδ^ 469 ± 27.4
1 h		^β^ 455 ± 13.1	435 ± 14.1	^β^ 440 ± 18.6	^αβ^ 465 ± 19.4	^αβγδ^ 477 ± 17.2	^αβγδ^ 475 ± 29.3
2 h		^β^ 460 ± 19.5	440 ± 20.2	^β^ 460 ± 24.3	^αβ^ 470 ± 21.3	^αβγδ^ 482 ± 19.4	^αβγδ^ 480 ± 24.6
3 h		^β^ 470 ± 21.4	450 ± 24.2	^β^ 470 ± 27.5	^αβ^ 480 ± 21.6	^αβγδ^ 490 ± 30.2	^αβγδ^ 483 ± 21.6
4 h		^β^ 455 ± 21.7	435 ± 22.4	^β^ 455 ± 15.1	^αβ^ 470 ± 15.9	^αβγδ^ 480 ± 25.4	^αβγδ^ 485 ± 31.7
5 h		^β^ 430 ± 16.1	425 ± 11.5	^αβ^ 445 ± 9.53	^αβ^ 450 ± 15.6	^αβγδ^ 475 ± 19.8	^αβγδ^ 480 ± 21.8
6 h		^β^ 422 ± 6.50	419 ± 7.25	^αβ^ 430 ± 10.7	^β^ 425 ± 13.3	^αβγδ^ 465 ± 26.5	^αβγδ^ 470 ± 24.6

**Table A4:** Time-course (dose 1200 mg) studies of intestinal barrier integrity on Transwell^®^ by TEER measurements. α *p* < 0.05 vs. Commercial product; β *p* < 0.05 vs. PEAm; γ *p* < 0.05 vs. PEAum; δ *p* < 0.05 vs. PEA 80 mesh.

	Sample	Commercial Product 1200 mg	PEAm 1200 mg	PEAum 1200 mg	PEA80mesch 1200 mg	PEA 1200 mg Ω	PEA 1200 mg DynoΩ
Time							
0.5 h		^β^ 450 ± 17.3	415 ± 9.78	^β^ 425 ± 9.54	^β^ 449 ± 19.4	^αβγδ^ 460 ± 10.2	^αβγδ^ 459 ± 12.9
1 h		^β^ 450 ± 13.6	425 ± 10.1	^β^ 430 ± 23.2	^αβ^ 455 ± 21.7	^αβγδ^ 467 ± 9.88	^αβγδ^ 465 ± 14.3
2 h		^β^ 455 ± 14.2	430 ± 11.8	^β^ 450 ± 26.5	^αβ^ 460 ± 28.1	^αβγδ^ 472 ± 13.9	^αβγδ^ 470 ± 10.4
3 h		^β^ 460 ± 19.7	440 ± 15.3	^β^ 455 ± 23.2	^αβ^ 470 ± 24.2	^αβγδ^ 480 ± 31.4	^αβγδ^ 473 ± 20.5
4 h		^β^ 445 ± 21.1	425 ± 16.2	^β^ 440 ± 26.7	^αβ^ 460 ± 26.3	^αβγδ^ 470 ± 14.2	^αβγδ^ 475 ± 21.3
5 h		^β^ 420 ± 17.7	415 ± 6.49	^αβ^ 435 ± 17.1	^αβ^ 440 ± 14.2	^αβγδ^ 465 ± 13.8	^αβγδ^ 470 ± 21.4
6 h		^β^ 412 ± 8.69	409 ± 4.56	^αβ^ 420 ± 12.8	^β^ 415 ± 11.3	^αβγδ^ 455 ± 12.7	^αβγδ^ 460 ± 19.5

**Table A5:** Time-course (dose 1800 mg) studies of intestinal barrier integrity on Transwell^®^ by TEER measurements. α *p* < 0.05 vs. Commercial product; β *p* < 0.05 vs. PEAm; γ *p* < 0.05 vs. PEAum; δ *p* < 0.05 vs. PEA 80 mesh.

	Sample	Commercial Product 1800 mg	PEAm 1800 mg	PEAum 1800 mg	PEA80mesch 1800 mg	PEA 1800 mg Ω	PEA 1800 mg DynoΩ
Time							
0.5 h		^β^ 420 ± 7.82	415 ± 8.71	418 ± 8.14	^αβγ^ 425 ± 12.3	^αβγδ^ 437 ± 14.3	^αβγδ^ 430 ± 12.6
1 h		^β^ 425 ± 9.23	420 ± 4.58	^αβ^ 430 ± 15.2	^αβ^ 430 ± 24.5	^αβγδ^ 442 ± 11.6	^αβγδ^ 435 ± 16.7
2 h		^β^ 435 ± 13.5	430 ± 14.5	^αβ^ 440 ± 21.5	^αβ^ 440 ± 13.1	^αβγ^ 445 ± 13.7	^αβγ^ 440 ± 26.1
3 h		^β^ 420 ± 8.25	415 ± 11.5	^αβ^ 425 ± 10.3	^αβγ^ 430 ± 17.2	^αβγδ^ 440 ± 21.3	^αβγδ^ 445 ± 24.8
4 h		^β^ 410 ± 6.54	405 ± 2.56	^αβδ^ 420 ± 9.89	^αβ^ 415 ± 8.94	^αβγδ^ 435 ± 23.4	^αβγδ^ 440 ± 20.9
5 h		^β^ 406 ± 3.46	404 ± 1.56	^αβ^ 415 ± 6.41	^αβ^ 411 ± 16.8	^αβγδ^ 425 ± 12.4	^αβγδ^ 430 ± 11.2
6 h		^β^ 420 ± 8.51	415 ± 4.23	418 ± 7.98	^αβγ^ 425 ± 11.5	^αβγδ^ 437 ± 11.4	^αβγδ^ 430 ± 17.1
